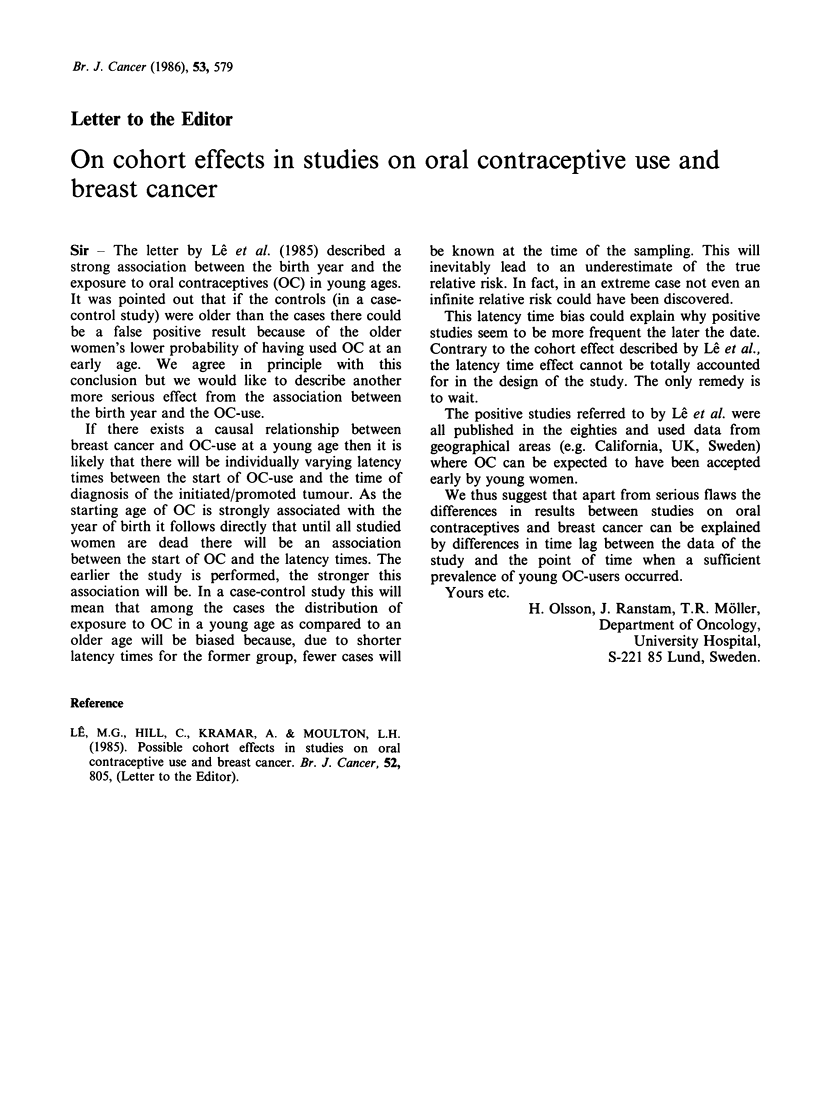# On cohort effects in studies on oral contraceptive use and breast cancer.

**DOI:** 10.1038/bjc.1986.92

**Published:** 1986-04

**Authors:** H. Olsson, J. Ranstam, T. R. Möller


*Br. J. Cancer* (1986), **53**, 579

### Letter to the Editor

### On cohort effects in studies on oral contraceptive use and breast cancer

**Sir** – The letter by Lê *et al.* (1985) described a strong association between the birth year and the exposure to oral contraceptives (OC) in young ages. It was pointed out that if the controls (in a case-control study) were older than the cases there could be a false positive result because of the older women's lower probability of having used OC at an early age. We agree in principle with this conclusion but we would like to describe another more serious effect from the association between the birth year and the OC-use.

If there exists a causal relationship between breast cancer and OC-use at a young age then it is likely that there will be individually varying latency times between the start of OC-use and the time of diagnosis of the initiated/promoted tumour. As the starting age of OC is strongly associated with the year of birth it follows directly that until all studied women are dead there will be an association between the start of OC and the latency times. The earlier the study is performed, the stronger this association will be. In a case-control study this will mean that among the cases the distribution of exposure to OC in a young age as compared to an older age will be biased because, due to shorter latency times for the former group, fewer cases will be known at the time of the sampling. This will inevitably lead to an underestimate of the true relative risk. In fact, in an extreme case not even an infinite relative risk could have been discovered.

This latency time bias could explain why positive studies seem to be more frequent the later the date. Contrary to the cohort effect described by Lê *et al.*, the latency time effect cannot be totally accounted for in the design of the study. The only remedy is to wait.

The positive studies referred to by Lê *et al.* were all published in the eighties and used data from geographical areas (e.g. California, UK, Sweden) where OC can be expected to have been accepted early by young women.

We thus suggest that apart from serious flaws the differences in results between studies on oral contraceptives and breast cancer can be explained by differences in time lag between the data of the study and the point of time when a sufficient prevalence of young OC-users occurred.

Yours etc.

H. Olsson, J. Ranstam, T.R. Möller,
Department of Oncology,
University Hospital,
S-221 85 Lund, Sweden.

### Reference

LÊ, M.G., HILL, C., KRAMAR, A. & MOULTON, L.H. (1985). Possible cohort effects in studies on oral contraceptive use and breast cancer. *Br. J. Cancer*, **52**, 805, (Letter to the Editor).